# Association between physical activity domains and depressive symptoms among Brazilian adults: does every move count?

**DOI:** 10.1590/0102-311XEN095723

**Published:** 2024-03-11

**Authors:** Mathias Roberto Loch, Nathalia Assis Augusto, Bruna Leticia Scremin Souza, Jessica Vertuan Rufino, Fabio Fortunato Brasil de Carvalho

**Affiliations:** 1 Universidade Estadual de Londrina, Londrina, Brasil.; 2 Instituto Nacional de Câncer, Rio de Janeiro, Brasil.

**Keywords:** Health Promotion, Chronic Disease, Life Style, Depression, Health Surveys, Promoção da Saúde, Doença Crônica, Estilo de Vida, Depressão, Inquéritos Epidemiológicos, Promoción de la Salud, Enfermedad Crónica, Estilo de Vida, Depresión, Encuestas Epidemiológicas

## Abstract

This study aimed to investigate the practice of physical activities in the four domains (leisure time, transportation, household, and work) and the prevalence of depressive symptoms in Brazilian adults, in general and stratified by sex, schooling level, and having or not a self-reported diagnosis of depression. This is a cross-sectional study with data from 88,531 individuals aged 18 years or older, who responded to the *Brazilian National Health Survey* in 2019. The depressive symptoms were evaluated by the *Patient Health Questionnaire-9* (PHQ-9). Those who practice physical activities at least once a week in a given domain were considered physically active. Additionally, the calculation of physical activities duration was conducted and later divided into quartiles for each domain. For the association analyses, the crude odds ratio (_crude_OR) and adjusted odds ratio (_adjusted_OR) were calculated for the total and stratified analyses. Individuals who are physically active during leisure time showed a lower chance of presenting depressive symptoms, in total (_adjusted_OR = 0.74; 95%CI: 0.64-0.86) and in all stratifications, except for individuals with self-reported depression. The associations of leisure-time physical activity were most frequent in those who practice from 121 to 360 minutes/week. The individuals who were active in the transportation, household, and work domains had a higher chance of presenting depressive symptoms in some groups, with more consistent results for household physical activities. The results showed that the relationship between physical activities and depression among Brazilians varies according to domain and duration, and that the concept that “every move counts” seemed to be correct only for the leisure-time domain.

## Resumo

O objetivo desta pesquisa foi investigar a relação da prática de atividade física nos quatro domínios (tempo livre, deslocamento, doméstico e trabalho) e a prevalência de sintomas depressivos em adultos brasileiros, de maneira geral e estratificando-se por sexo, escolaridade e ter ou não diagnóstico referido de depressão. Estudo transversal, com dados de 88.531 indivíduos de 18 anos ou mais, respondentes da *Pesquisa Nacional de Saúde* de 2019. Os sintomas depressivos foram avaliados pelo *Patient Health Questionnaire-9* (*Questionário de Saúde do Paciente-9*, PHQ-9). Foram considerados fisicamente ativos aqueles que referiram realizar atividade física pelo menos uma vez por semana no respectivo domínio. Adicionalmente, foi realizado o cálculo de tempo de prática semanal, sendo posteriormente divididos em quartis em cada domínio. Para as análises de associação, foram calculados o *odds ratio* bruto (OR_bruto_) e ajustado (OR_ajustado_), no total e nas análises estratificadas. Os fisicamente ativos no tempo livre tiveram menor chance de apresentar sintomas depressivos, no total (OR_ajustado_ = 0,74; IC95%: 0,64-0,86) e em todas as estratificações, menos naqueles com depressão autorreferida. As associações na atividade física no tempo livre foram mais frequentes naqueles que praticavam entre 121 e 360 minutos semanais. Os indivíduos ativos nos domínios de deslocamento, doméstico e trabalho tiveram maior chance de apresentar sintomas depressivos em alguns grupos, com resultados mais consistentes para a atividade física doméstica. Os resultados evidenciaram que a relação da atividade física com a depressão em brasileiros varia conforme o domínio e a duração da atividade física, e que a ideia de que “todo movimento conta” parece adequada apenas para o domínio de tempo livre.

## Resumen

Este estudio tuvo como objetivo investigar la práctica de actividad física en cuatro dominios (ocio, desplazamiento, actividad doméstica y trabajo) y la prevalencia de síntomas depresivos en adultos brasileños, en general y estratificada por sexo, escolaridad y diagnóstico de depresión autoinformado. Se trata de un estudio transversal con datos de 88.531 individuos de 18 años o más, que respondieron la *Encuesta Nacional de Salud* en el 2019. Los síntomas depresivos se evaluaron mediante el *Cuestionario sobre la Salud del Paciente-9* (PHQ-9). Aquellos que realizan actividad física al menos una vez por semana en un dominio determinado se consideraron físicamente activos. Además, se calculó el tiempo de actividad física y luego se dividió en cuartiles para cada dominio. Para los análisis de asociación, se calcularon el *odds ratio* crudo (OR_crudo_) y el *odds ratio* ajustado (OR_ajustado_) para los análisis total y estratificado. Los individuos que son físicamente activos en durante el ocio presentaron menos probabilidades de tener síntomas depresivos, en el total (OR_ajustado_ = 0,74; IC95%: 0,64-0,86) y en todas las estratificaciones, excepto los individuos con depresión autoinformada. Las asociaciones de actividad física en el tiempo libre fueron más frecuentes en quienes practicaban de 121 a 360 minutos/semana. Los individuos que eran activos en los dominios desplazamiento, actividad doméstica y trabajo tuvieron más probabilidades de presentar síntomas depresivos en algunos grupos, con resultados más consistentes para las actividades domésticas. Los resultados mostraron que la relación entre actividad física y depresión entre los brasileños varía según el dominio y la duración, y el concepto de que “cada movimiento cuenta” parece ser correcto solo para el dominio del ocio.

## Introduction

Major depressive disorder, also known as depression, represents a classic condition in the group of depressive disorders. Clinical depression symptoms include depressed humor, lack of interest, sleeping disorders, agitation or psychomotor delay, exhaustion, feeling worthless or excessive guilt, among others ^1^.

It is estimated that depression affects 350 million people worldwide ^2^. In Brazil, according to the *National Health Survey* 2019 (PNS 2019) ^3^, around 10% of all individuals reported a medical diagnosis of depression. This disorder causes important consequences, such as work absenteeism, high burden for the health system, as well as implications for social relationships ^4^.

Physical activity is indicated as an important health behavior, representing a protection factor against depression, and may also improve symptoms in those who are diagnosed. Therefore, physical activity has been recommended for general health and for preventing and treating depression ^5^ ^,^ ^6^ ^,^ ^7^ ^,^ ^8^.

Physically active Brazilians have less chance of presenting depression; however, the relationship between physical activity and depression varies according to the type of physical activity ^8^. For instance, activities in open and/or collective spaces may bring more benefits, since they are not only related to physiological mechanisms, but also to other contextual effects, such as greater social interaction, exposure to sunlight, and natural spaces, which can provide a more pleasant experience ^8^. However, the authors of this study analyzed only leisure-time activities.

Besides the leisure-time (free-time) domain, physical activity is classically divided into three other domains: transportation, work, and household ^7^ ^,^ ^9^. Generally, leisure-time physical activity is practiced during a time of enjoyment, often related to people’s choices and preferences. Meanwhile, work physical activity refers to the act of working; transportation physical activity is practiced as a means of active movement to go from one place to another, and finally, household physical activity is performed to take care of the home and of the family, and includes, for instance, the tasks of cleaning and gardening ^7^.

Regarding the public health message that “every move counts” ^10^, we believe that it may be understood based on the evidence that every activity has potential benefits to health, regardless of how long they last and their intensity, and that no clear minimum limit is necessary to obtain some type of benefit, especially if one considers the multiple potential “outcomes” that physical activity may influence, differently from what has been proposed: such benefits only happen when based on official recommendations ^11^ ^,^ ^12^ ^,^ ^13^.

However, the literature presents an ongoing discussion, which can be called the paradox of physical activity ^14^, indicating that the relationship of physical activity with health indicators may depend on which type of physical activity is performed. Generally, such a “paradox” has indicated that, if on the one hand leisure-time physical activity is associated with better health indicators, in other domains, particularly the work domain, physical activity may have negative associations, depending on specific features, such as staying in an inadequate posture for long periods, heavy lifting, and a lack of sufficient recovery time ^15^ ^,^ ^16^ ^,^ ^17^. Overall, the studies related to the referred paradox focused on general mortality and cardiovascular diseases ^17^ ^,^ ^18^ as well as on absenteeism ^15^.

Regarding the association between the different physical activity domains and depressive symptoms, a previous study identified that such association was non-linear for all physical activity domains, and it was observed that the practice of leisure-time physical activity, even at lower levels, was associated with a lower prevalence of depressive symptoms, while that result was not observed in other domains, with some specificities depending on the age ^19^. For instance, in the younger participants (15-24 years of age), transportation physical activity was associated with a lower prevalence of depressive symptoms, whereas the opposite was found for the oldest individuals (65 years or more) ^19^. The study ^19^ conducted overall analyses considering the entire sample, including individuals aged 15 and older, and stratified the data by age group, but it did not investigate whether theor associations varied according to gender, educational level, or the presence of self-reported depression diagnosis.

Considering this, a more specific understanding of the relationship between different physical activity domains with depression may be important, even when contemplating more effective actions for the prevention and treatment of the condition, as well as for planning actions and public policies that aim to promote health. This study’s hypothesis is that associations may vary across different physical activity domains and group characteristics, i.e., they may differ according to gender, educational level, and among individuals with or without a diagnosis of depression. Therefore, this study aimed to investigate the relationships of physical activity in its four domains (leisure time, transportation, household, and work) and the prevalence of depressive symptoms in Brazilian adults, both overall and with the sample stratified by sex, schooling level, and presence or not of a self-reported depression diagnosis.

## Methodology

### Study design and population

This is a cross-sectional study, based on data from the PNS 2019, which is a household population-based survey aiming to produce data on access to and use of health services, and the health situation of the Brazilian population ^3^. The PNS 2019 was approved by the Institutional Review Board of the Brazilian National Research Ethics Commitee (CONEP, opinion n. 3,529,376).

This study considered 88,531 adults, aged 18 years and older, who answered an individual questionnaire ^20^. A more detailed description of the PNS methodology can be found in previous publications ^3^ ^,^ ^21^.

### Variables

The dependent variable in this study were the depression symptoms, evaluated by the *Patient Health Questionnaire-9* (PHQ-9), with a scale adapted and validated for the Brazilian population ^22^. A study carried out with data from the PNS 2019 showed good psychometric properties for the PHQ-9, demonstrated by the good fit of data for the unidimensional model (root mean square error of approximations - RMSEA = 0.060; comparative fit index - CFI = 0.992; Tucker-Lewis index - TLI = 0.989; standardized root means square residuals - SRMR = 0.052), adequate internal consistency (ω = 0.875), and significant invariance across sexes, ages, and Brazilian states ^23^. The instrument includes nine questions that evaluate the presence and the level of depression, based on diagnostic criteria from the *Diagnostic and Statistical Manual of Mental Disorders* (DSM), based on one’s self-perception of the symptoms in the previous two weeks. Each question has an answer that ranges from 0-3; therefore, the sum may reach 27 points. Individuals with a score ≥ 10 were considered to have depressive symptoms ^24^.

The four physical activities domains were the independent variables. Individuals were considered to be active when the following activities were reported at least once a week: leisure-time physical activity (walking, cycling, dancing, or sports like soccer and volleyball, among others); transportation physical activity (going to and from work, school, or club by bicycle or walking, even if only as company for another person, not considering work situations); household physical activity (heavy cleaning, carrying weights, or any other heavy household activity that requires intense effort, not considering paid activities); work physical activity (walking around at work and/or performing heavy cleaning, carrying weights, or any other heavy activity which requires intense physical effort in the work environment). For work physical activity, only individuals with an occupation were included in the study.

Individuals considered active in each domain informed the amount of time they spent on a usual day practicing physical activity. The time reported was multiplied by the weekly frequency, resulting in the duration of the weekly physical activity practice (in minutes). For leisure-time physical activity, weights were attributed according to the intensity of the activity. In that case, moderate physical activity weighted ^1^ and vigorous physical activity weighted ^2^ in the calculation of time, with the intensities having been previously defined by the PNS 2019 ^3^. The physical activity in minutes/week was divided into quartiles, considering, for that purpose, only subjects which indicated some practice in the respective domain. For leisure-time physical activity and household physical activity, the quartiles represent the same values: Q1 (1-120 minutes), Q2 (121-240 minutes), Q3 (241-360 minutes), and Q4 (≥ 361 minutes). For transportation physical activity the quartiles are: Q1 (1-60 minutes), Q2 (61-120 minutes), Q3 (121-240 minutes), and Q4 (≥ 241 minutes). For work physical activity the quartiles are: Q1 (1-350 minutes), Q2 (351-900 minutes), Q3 (901-1,749 minutes), and Q4 (≥ 1,750 minutes).

Sociodemographic characteristics, included as confounding variables, and their respective categories were: sex (men; women), age group in years (18-39; 40-59; 60 or more), marital status (with or without spouse), race/skin color (white/yellow; black/mixed-race/Indigenous), educational level (complete college or more; complete high school; illiterate to complete elementary school), household income per capita in minimum wages (> 4.5; > 2.0-4.5; > 1.0-2.0; 0.0-0.5). The variables related to depression treatment were confounding variables as well, specifically: medication treatment, psychotherapy treatment, and use of complementary integrative practices, with the same characterization for the three variables (depression diagnosis: with treatment; without treatment; and without depression diagnosis). Moreover, the body mass index (BMI) (low weight; eutrophic; overweight; and obesity) and the practice in the four physical activity domains (yes; no) were also included, except when the domain was the independent variable (for instance, when leisure-time physical activity was the independent variable, the other domains were considered as the confounding variables).

Moreover, the analyses were stratified by sex (men; women), educational level (complete college; complete high school; illiterate to complete elementary school), and self-reported depression diagnosis (separated into “reported” and “not reported” previous depression diagnosis by a physician or mental health professional).

### Data processing and analysis

For sample characterization, the prevalence of the sociodemographic characteristics was calculated, as was the practice of physical activity in the four domains, and the depressive symptoms according to PHQ-9 and self-reported depression. The analyses of the association between physical activity in the four domains and the depressive symptoms were conducted based on the crude and adjusted odds ratios (_crude_OR/_adjusted_OR) and 95% confidence intervals (95%CI), presented in total and stratified by sex, educational level, and presence or not of a self-reported depression diagnosis. To verify the association between the amount of time practicing physical activity in the four domains and depressive symptoms, the _adjusted_OR and the 95%CI were calculated, and presented in both total and stratified by sex, educational level, and self-reported depression diagnosis. The reference category for the association analyses was always the group that did not practice physical activity in each domain. All analyses were conducted with the IBM SPSS program, version 19.0 (https://www.ibm.com/), considering the sample weighting of the PNS 2019.

## Results

Data from 88,531 individuals, aged 18 years and older, were analyzed (53.2% were women). A higher frequency was observed for individuals in the age group from 19-39 years (43.1%); without a spouse (56.1%); who declared themselves black, mixed-race, or Indigenous (59.7%); without a complete college (84.2%); and with household income per capita of up to one minimum wage (51.3%). The prevalence of physical activity practice in the four domains, regardless of the length, was 40.5% in the leisure-time, 49.6% in the transportation, 15.8% in the household, and 48% in the work domain. Regarding depressive symptoms, the prevalence was 10.8% according to PHQ-9, and 10.2% reported having been diagnosed with depression (Table 1).

**Table 1:** Characterization of the sample of adults, aged 18 years or older. *Brazilian National Health Survey*, 2019 (n = 88,531 *).

Characteristics	n	% **
Sex		
Men	41,662	46.8
Women	46,869	53.2
Age group (years)		
18-39	33,544	43.1
40-59	32,259	35.3
60 or more	22,728	21.6
Marital status		
With spouse	35,110	43.9
Without spouse	53,421	56.1
Race/Skin color ***		
White/Yellow	33,074	40.3
Black/Mixed-race/Indigenous	55,448	59.7
Education level		
Complete college or more	13,617	15.8
Complete high school	27,337	34.9
Illiterate to complete elementary school	47,577	49.3
Income (minimum wage)		
> 4.5	5,754	6,0
> 2.0-4.5	12,295	14.6
> 1.0-2.0	22,153	28.2
> 0.5-1.0	25,657	29.1
0.0-0.5	22,646	22.2
Leisure-time physical activity		
Yes	33,739	40.5
No	54,792	59.5
Transportation physical activity		
Yes	42,272	49.6
No	46,259	50.4
Household physical activity		
Yes	12,912	15.8
No	75,619	84.2
Work physical activity ^#^		
Yes	25,400	48,0
No	27,075	52,0
Depressive symptoms		
Yes	9,252	10.8
No	79,279	89.2
Self-reported depression diagnosis		
Yes	8,242	10.2
No	80,289	89.8

* n weighted = 159,171,311;

** The frequencies presented are weighted;

*** Nine individuals are missing information on race/skin color;

^#^ 36,056 individuals with no occupation were not included in the analysis of work physical activity.

Regarding the association between physical activity domains and depressive symptoms, we observed that the subjects who practiced physical activity in their leisure time had a lower chance of presenting depressive symptoms, generally (_adjusted_OR = 0.74; 95%CI: 0.64-0.86) and in both sexes. By contrast, in the household physical activity domain, those who practiced physical activity had a higher chance of presenting depressive symptoms (_adjusted_OR = 1.38; 95%CI: 1.18-1.62), given that the same was observed when the sample was stratified by sex, even though the OR observed for men (_adjusted_OR = 1.83; 95%CI: 1.31-2.54) was higher than that for women (_adjusted_OR = 1.25; 95%CI: 1.06-1.49), indicating a stronger relationship in the case of men. Transportation PA was associated only in the case of men (_adjusted_OR = 1.37; 95%CI: 1.10-1.70), and the chance of presenting depressive symptoms was higher among those who are active in that domain. Work physical activity was associated, in general (_adjusted_OR = 1.17; 95%CI: 1.01-1.35) and in women (_adjusted_OR = 1.35; 95%CI: 1.13-1.62), with a higher chance of depressive symptoms among those who are active in this domain (Table 2).

**Table 2:** Association between practice of physical activities in the four domains and depressive symptoms in men and women, aged 18 years and older, with different levels of education. *Brazilian National Health Survey*, 2019 (n = 88,531).

Domain of physical activity	Depressive symptoms					
	Total		Men		Women	
	_crude_ **OR (95%CI)**	_adjusted_ OR * (95%CI)	_crude_ **OR (95%CI)**	_adjusted_ OR ** (95%CI)	_crude_ **OR (95%CI)**	_adjusted_ OR ** (95%CI)
Leisure time						
No	1.00	1.00	1.00	1.00	1.00	1.00
Yes	0.60 (0.55-0.65)	0.74 (0.64-0.86)	0.60 (0.50-0.71)	0.75 (0.60-0.94)	0.65 (0.59-0.72)	0.76 (0.64-0.91)
Transportation						
No	1.00	1.00	1.00	1.00	1.00	1.00
Yes	1.04 (0.96-1.13)	1.13 (0.98-1.29)	1.10 (0.94-1.28)	1.37 (1.10-1.70)	0.97 (0.88-1.07)	0.97 (0.82-1.15)
Household						
No	1.00	1.00	1.00	1.00	1.00	1.00
Yes	1.65 (1.51-1.80)	1.38 (1.18-1.62)	1.55 (1.24-1.94)	1.83 (1.31-2.54)	1.32 (1.19-1.46)	1.25 (1.06-1.49)
Work						
No	1.00	1.00	1.00	1.00	1.00	1.00
Yes	1.10 (0.98-1.24)	1.17 (1.01-1.35)	1.01 (0.82-1.25)	0.97 (0.78-1.20)	1.47 (1.27-1.70)	1.35 (1.13-1.62)

95%CI: 95% confidence interval; OR: odds ratio.

* Adjusted by sociodemographic variables (sex, age group, race/skin color, marital status, education, and income), domains of physical activity, depression treatment (medication treatment, psychotherapeutic treatment, and use of complementary integrative practices) and body mass index;

** Adjusted by the same variables in the total (*), without the variable sex.

In the analysis stratified by educational level, the leisure-time domain was associated with the three categories of education, and a lower chance of depressive symptoms was observed among the physically active. The opposite was found in the household physical activity domain, with a higher chance of depressive symptoms for those who are active in that domain in the categories of complete secondary and complete college or more. Regarding transportation domain, an association was observed only for the group with a lower level of education, also showing a higher chance of depression among the most active. Regarding work, significant associations were observed for individuals with higher and lower educational levels, where those active in this domain had a greater chance of presenting depressive symptoms (Table 3).

**Table 3:** Association between practice of physical activities in the four domains and depressive symptoms in adults, aged 18 years and older, with different levels of education. *Brazilian National Health Survey*, 2019 (n = 88,531).

Domain of physical activity	Depressive symptoms					
	Illiterate to complete elementary school		Complete high school		Complete college or more	
	_crude_ **OR (95%CI)**	_adjusted_ OR * (95%CI)	_crude_ **OR (95%CI)**	_adjusted_ OR * (95%CI)	_crude_ **OR (95%CI)**	_adjusted_ OR * (95%CI)
Leisure time						
No	1.00	1.00	1.00	1.00	1.00	1.00
Yes	0.65 (0.57-0.73)	0.79 (0.64-0.99)	0.65 (0.56-0.75)	0.73 (0.59-0.91)	0.51 (0.41-0.64)	0.64 (0.47-0.89)
Transportation						
No	1.00	1.00	1.00	1.00	1.00	1.00
Yes	1.00 (0.91-1.11)	1.31 (1.08-1.60)	1.17 (1.01-1.35)	1.11 (0.89-1.38)	0.88 (0.70-1.11)	0.89 (0.65-1.22)
Household						
No	1.00	1.00	1.00	1.00	1.00	1.00
Yes	1.43 (1.26-1.62)	1.21 (0.94-1.55)	1.93 (1.64-2.26)	1.48 (1.17-1.87)	2.04 (1.61-2.58)	1.53 (1.04-2.25)
Work						
No	1.00	1.00	1.00	1.00	1.00	1.00
Yes	1.12 (0.94-1.32)	1.29 (1.05-1.59)	0.91 (0.76-1.09)	0.95 (0.78-1.17)	1.48 (1.08-2.04)	1.51 (1.03-2.20)

95%CI: 95% confidence interval; OR: odds ratio.

* Adjusted by sociodemographic variables (sex, age group, race/color of skin, marital status, and income), domains of physical activity, depression treatment (medication treatment, psychotherapeutic treatment, and use of complementary integrative practices), and body mass index.

When the analysis was conducted, stratified by having or not having a diagnosis of depression, it showed that, among those with the diagnosis, only the household domain had an association with a higher chance of depressive symptoms among those physically active in this domain (_adjusted_OR = 1.30; 95%CI: 1.00-1.68). Among those without this diagnosis, all physical activity domains showed an association. However, while the leisure-time physical activity practice decreased the chance of presenting depressive symptoms, in the other domains, the practice increased that chance (Table 4).

**Table 4:** Association between practice of physical activities in the four domains and depressive symptoms in adults, 18 years of age and older, with and without self-reported depression. *Brazilian National Health Survey*, 2019 (n = 88,531).

Domain of physical activity	Depressive symptoms		
	With self-reported depression diagnosis		Without self-reported depression diagnosis	
	_crude_ **OR (95%CI)**	_adjusted_ OR * (95%CI)	_crude_ **OR (95%CI)**	_adjusted_ OR ** (95%CI)
Leisure time				
No	1.00	1.00	1.00	1.00
Yes	0.67 (0.56-0.79)	0.80 (0.60-1.05)	0.56 (0.50-0.62)	0.74 (0.62-0.87)
Transportation				
No	1.00	1.00	1.00	1.00
Yes	0.93 (0.80-1.09)	0.91 (0.71-1.17)	1.11 (1.00-1.22)	1.19 (1.01-0.139)
Household				
No	1.00	1.00	1.00	1.00
Yes	1.10 (0.90-1.33)	1.30 (1.00-1.68)	1.72 (1.54-1.92)	1.42 (1.17-1.72)
Work				
No	1.00	1.00	1.00	1.00
Yes	1.06 (0.85-1.33)	0.99 (0.77-1.28)	1.22 (1.06-1.40)	1.26 (1.07-1.48)

95%CI: 95% confidence interval; OR: odds ratio.

* Adjusted by sociodemographic variables (sex, age group, race/skin color, marital status, education, and income), domains of physical activity, depression treatment (medication treatment, psychotherapeutic treatment, and use of complementary integrative practices), and body mass index;

** Adjusted by sociodemographic variables (sex, age group, race/skin color, marital status, education, and income), domains of physical activity, and body mass index.

When the analyses of the physical activity domains were conducted based on quartiles or weekly physical activity practice, with the subjects who did not practice physical activity as the reference group, a non-linear relationship was observed during leisure time, especially given the results in the most active quartile. Most of the associations were observed in quartiles 2 and 3 (121-240 minutes/week and 241-360 minutes/week, respectively). In the comparison of quartile 1 (1-120 minutes/week) with the reference group, only one association was observed (in those with complete high school), but note that in all stratifications tested, the OR value was below 100 in the subjects of quartile 1 (Table 5).

**Table 5:** Association between the amount of time (in minutes) of weekly practice of physical activities in the four domains and depressive symptoms in adults, aged 18 years and older. *Brazilian National Health Survey*, 2019 (n = 88,531).

Amount of physical activity time (minutes) in the four domains	Depressive symptoms							
	Total	Women	Men	Illiterate to complete elementary school	Complete high school	Complete college or more	With self-reported depression	Without self-reported depression
	_adjusted_ OR * (95%CI)	_adjusted_ OR ** (95%CI)	_adjusted_ OR ** (95%CI)	_adjusted_ OR *** **(95%CI)**	_adjusted_ OR *** (95%CI)	_adjusted_ OR *** (95%CI)	_Adjusted_ OR * (95%CI)	_adjusted_ **OR** ^#^ **(95%CI)**
Leisure time								
Does not practice	1.00	1.00	1.00	1.00	1.00	1.00	1.00	1.00
Q1 (1-120)	0.80 (0.61-1.03)	0.80 (0.59-1.08)	0.88 (0.60-1.28)	0.85 (0.61-1.17)	0.66 (0.45-0.98)	0.85 (0.50-1.45)	0.79 (0.53-1.19)	0.81 (0.61-1.09)
Q2 (121-240)	0.67 (0.53-0.84)	0.72 (0.53-0.97)	0.63 (0.46-0.86)	0.59 (0.41-0.83)	0.71 (0.47-1.05)	0.62 (0.43-0.91)	0.64 (0.44-0.93)	0.70 (0.53-0.92)
Q3 (241-360)	0.71 (0.57-0.90)	0.75 (0.56-0.99)	0.69 (0.49-0.99)	1.02 (0.68-1.51)	0.68 (0.48-0.97)	0.51 (0.32-0.80)	0.82 (0.49-1.37)	0.69 (0.54-0.88)
Q4 (≥ 360)	0.79 (0.63-1.00)	0.79 (0.59-1.06)	0.80 (0.56-1.16)	0.82 (0.55-1.21)	0.89 (0.63-1.25)	0.57 (0.35-0.93)	1.01 (0.64-1.59)	0.74 (0.57-0.98)
Transportation								
Does not practice	1.00	1.00	1.00	1.00	1.00	1.00	1.00	1.00
Q1 (1-60)	1.08 (0.89-1.31)	0.97 (0.77-1.22)	1.19 (0.85-1.67)	1.24 (0.93-1.64)	1.03 (0.74-1.44)	0.86 (0.58-1.28)	1.14 (0.81-1.60)	1.03 (0.81-1.30)
Q2 (61-120)	1.01 (0.83-1.23)	0.90 (0.71-1.14)	1.20 (0.88-1.64)	1.11 (0.82-1.50)	1.10 (0.80-1.50)	0.79 (0.52-1.20)	0.85 (0.58-1.25)	1.06 (0.84-1.33)
Q3 (121-240)	1.20 (0.97-1.47)	0.97 (0.76-1.24)	1.65 (1.18-2.31)	1.50 (1.10-1.05)	1.11 (0.79-1.54)	0.86 (0.52-1.42)	0.69 (0.48-1.00)	1.36 (1.08-1.74)
Q4 (≥ 241)	1.24 (1.01-1.53)	1.07 (0.84-1.38)	1.45 (1.05-2.00)	1.40 (1.06-1.84)	1.22 (0.86-1.71)	1.19 (0.73-1.92)	0.95 (0.63-1.43)	1.33 (1.06-1.65)
Household								
Does not practice	1.00	1.00	1.00	1.00	1.00	1.00	1.00	1.00
Q1 (1-120)	1.23 (0.92-1.65)	1.11 (0.80-1.54)	1.46 (0.81-2.63)	0.82 (0.55-1.21)	1.36 (0.93-2.00)	1.83 (0.92-3.64)	1.04 (0.65-1.67)	1.26 (0.88-1.80)
Q2 (121-240)	1.16 (0.88-1.52)	1.00 (1.75-1.33)	2.08 (1.17-3.69)	0.79 (0.52-1.22)	1.53 (1.03-2.29)	1.26 (0.74-2.14)	0.89 (0.57-1.41)	1.31 (0.97-1.78)
Q3 (241-360)	1.51 (1.13-2.02)	1.37 (1.01-1.85)	2.14 (0.98-4.67)	2.02 (1.30-3.14)	1.15 (0.69-1.91)	1.61 (0.93-2.80)	1.90 (1.12-3.25)	1.38 (0.99-1.94)
Q4 (≥ 360)	1.74 (1.35-2.25)	1.60 (1.22-2.10)	2.26 (1.32-3.86)	2.02 (1.36-3.02)	1.83 (1.27-2.63)	1.44 (0.77-2.72)	1.70 (1.04-2.77)	1.77 (1.35-2.31)
Work								
Does not practice	1.00	1.00	1.00	1.00	1.00	1.00	1.00	1.00
Q1 (1-350)	1.01 (0.83-1.24)	1.13 (0.88-1.44)	0.89 (0.63-1.25)	1.24 (0.91-1.67)	0.73 (0.53-1.00)	1.18 (0.76-1.82)	0.89 (0.61-1.30)	1.07 (0.85-1.35)
Q2 (351-900)	0.96 (0.78-1.17)	1.13 (0.88-1.44)	0.75 (0.54-1.03)	0.95 (0.73-1.25)	0.87 (0.62-1.21)	1.36 (0.91-2.04)	0.96 (0.64-1.44)	0.98 (0.79-1.22)
Q3 (901-1,749)	1.55 (1.18-2.04)	1.74 (1.22-2.50)	1.35 (0.91-2.01)	1.36 (0.98-1.89)	1.38 (0.99-1.91)	2.88 (1.28-6.50)	1.26 (0.85-1.89)	1.69 (1.22-2.33)
Q4 (≥ 1,750)	1.28 (1.05-1.57)	1.60 (1.24-2.08)	0.98 (0.73-1.31)	1.54 (1.17-2.02)	0.96 (0.71-1.30)	1.19 (0.73-1.96)	0.94 (0.63-1.40)	1.41 (1.13-1.75)

95%CI: 95% confidence interval; OR: odds ratio.

* by sociodemographic variables (sex, age group, race/skin color, marital status, and income), domains of physical activity, depression treatment (medication treatment, psychotherapeutic treatment, and use of complementary integrative practices), and body mass index;

** Adjusted by the same variables of the total (*) without the variable sex;

*** Adjusted by the same variables of the total (*) without the variable education;

^#^ Adjusted by the same variables of the total (*) without the depression treatment variables (medication treatment, psychotherapeutic treatment, and use of complementary integrative practices).

Regarding transportation physical activity, which is the domain with the most associations, considering the entire sample, a significant association was observed only in the last quartile (241 minutes/week or more), whereas the chance of depressive symptoms was higher for those who are more physically active in this domain. In the stratified analyses, the data obtained reinforce the nonlinearity and some specificities regarding the direction of the association. Depressive symptoms showed a higher chance in the two final quartiles (3 and 4) from the group of men, among those with up to primary education and in those with no self-reported depression. On the other hand, opposite results were observed in women, with a complete tertiary education and self-reported depression, indicating a protective relationship, although not significant (Table 5).

Regarding household domain, in general, higher associations were observed in quartiles 3 (241 to 360 minutes/week) and 4 (361 minutes/week or more), with associations in nearly all the groups, in at least one of the two quartiles. A lack of association was only observed in the group of those with complete tertiary education. In all the significant associations identified in this study, those who are physically active in this domain showed a greater chance of having depressive symptoms when compared with the reference group (Table 5).

Regarding work domain, a higher number of associations was also observed in quartiles 3 (901-1,749 minutes/week) and 4 (1,750 minutes/week or more), with all significant associations indicating a higher risk of depression for the physically active, compared with the reference group (Table 5).

## Discussion

Some of the main findings in this study are: (a) the relationship between physical activity and the depressive symptoms seem to vary according to the domain; (b) the leisure-time domain was associated with the lowest chances of depressive symptoms; moreover, but not linearly, the most evident relationship was found in time of practice from 121-360 minutes/week; (c) the other domains (transportation, household, and work), when they did show an association, which was opposite to that observed for the leisure-time domain, were associated with greater chances of depressive symptoms; (d) outside the domains associated with the higher risk of depressive symptoms, the household domain was the one with the most consistent results; (e) some specificities in associations were observed in the stratified analyses, indicating differences in associations between men and women, among educational levels, and between those who have a diagnosis of depression or not.

Regarding the possible mechanisms of the relationship between physical activity and depression, some neuromuscular mechanisms seem relevant, which may increase the expression of neurotrophic factors, as well as the regulation of the activity in the hypothalamus-hypophysis-adrenal axis; the availability of serotonin and norepinephrine; and the reduction of systemic inflammation ^25^. Furthermore, physical activity may contribute indirectly by promoting bodily changes that bring better self-esteem and lead to greater satisfaction and happiness ^26^. However, considering the results of this study, those mechanisms may not act in the same manner in all physical activity domains. The leisure-time domain was the only one that presented an evident protection to depressive symptoms, with significant associations being observed in all groups, except for the group of those with self-reported depression (and even so, with an OR = 0.80, with the highest 95%CI value relatively close to 1.00).

Part of these results may be explained by leisure-time physical activity being most often a chosen activity, in which individuals decide to participate, either by the satisfaction achieved, or by cultural influence. On the other hand, in the other domains, the concept of “choice” is not present in the same manner.

Moreover, the “distraction hypothesis” may be considered, which proposed that part of the improvements in mental well-being resulting from physical activity is due to the distraction from unpleasant thoughts and feelings during physical activities ^27^. Such a “distraction” is likely to be more evident in the leisure-time domain, and it may not occur at all in the other domains, especially in the household and work domains. We should also consider that social interaction plays a role in this relationship. The leisure-time physical activity often takes place in a pleasant social environment, and the improvements earned by this kind of activity are, at least partially, related to mutual support and social relationships that happen during physical activities involving other people ^28^.

Regarding the transportation domain, some considerations must be made, especially because this domain yielded the least conclusive findings. One factor to be considered is that while transportation domain showed a greater chance of depressive symptoms among the physically active who have a lower educational level, the association ceased to exist among those with an intermediate or higher education. Moreover, the OR values obtained in the adjusted analysis were 1.31, 1.11, and 0.89, respectively, for lower, intermediate, and higher education. These data indicate a different pattern regarding physical activity in this domain according to education, even related to the intentionality of the activities, especially when we consider the strong connection between education and income in Brazil. Hence, part of those with a higher education who practice physical activity in the transportation domain, may do so as an option, perhaps because they live closer to work. On the other hand, for those with a lower educational level, practicing physical activity in the transportation domain is not a matter of choice, but rather of necessity ^29^, due to not having their own motor vehicle, or needing to save money on the transportation. Moreover, this kind of physical activity may increase the chances of dealing with stress factors, like traffic, enhancing the negative relationship ^30^.

Further studies are necessary, especially to understand the perceptions and the meanings attributed to active transportation by people with different levels of education and income. We would like to highlight, however, that the issue of promoting physical activity by encouraging active transportation may make sense when conducted in an expanded perspective, which considers the environment, the rights to citizenship, and urban mobility, and not according to a perspective that merely encourages active transport-related among people who have little choice in the matter.

In the household domain, the data seem to strongly indicate that practicing household physical activity is a factor that increases the chances of depressive symptoms. Besides the issue of the lack of choice, we need to consider that household chores are often conducted as a “third shift”. Many people work all day and “accumulate” an additional shift when they get home. In Brazil, those with more chances of being active in the household domain are women, individuals who are Black, and those who have a spouse ^3^ ^,^ ^31^. Clearly, household chores are a type of work that should be more valued, but they should not be seen in a simplistic manner, according to the perspective that the energy spent is always or nearly always something positive for health.

In the work domain, although there were fewer associations in comparison to those of the household domain, risk associations were always present, which means that those physically active in this domain had a greater chance of presenting depression symptoms. Much like the household domain, the possible explanations are related to the issue of choice, i.e., they are not activities that people choose to do, but rather need to do. We must highlight that, in Brazil, there is an important symbolic difference between works considered as “intellectual” and as “physical”. One example is the physical activity practice in that domain being associated with income and education in a study conducted with Brazilian adults ^31^. Such difference includes considering intellectual work as more valuable, even in terms of remuneration; therefore, people prefer to have jobs that require less physical effort. One piece of information that stood out in our study was the significant association between the extreme education groups, but not in the intermediate group. Furthermore, a stronger association was also observed among those with a complete college or more (OR = 1.51, compared with OR = 1.29 among those with up to primary education).

Still regarding the specific results in the work domain, one important element is that the physical activity carried out in that specific context is usually made up of repetitive and simple tasks, which causes the activity to generate no “distraction”, when compared with activities performed in leisure time, and those activities may constitute another source of stress, which is consequently harmful to mental health ^17^. Furthermore, physical activities related to the work domain are also seen as mandatory tasks to be done to obtain an external reward (payment), and, in such cases, motivation is controlled ^30^. According to the self-determination theory, the behaviors conducted by autonomous motivation are related to the satisfaction of psychological needs (autonomy, competence, and relationship). Thus, the feeling of well-being is increased when the psychological needs of the individuals are fulfilled (which does not happen in mandatory activities with controlled motivation) ^32^.

Considering the differences between sexes, although associations were observed for both sexes in the leisure-time and household domains, the transportation domain was only associated with men, and the work domain, with women. Although associated for both sexes, the _adjusted_OR values obtained in this study - even in the analysis conducted by dividing into quartiles according to the amount of time spent practicing physical activity in each domain (Table 5) - in the household domain shows differences indicating a stronger relationship among women, reinforcing the concept of the previously mentioned “third shift”. In the stratifications by education, we have already mentioned important specificities related to the transportation and work domains; however, in the leisure-time and household domains, the results were similar.

The stratification according to being diagnosed or not with depression showed important specificities. The group with a diagnosis of depression was the subgroup which had the least number of associations with the physical activity domains. Generally, only the leisure-time domain showed an association, and when divided into quartiles of physical activity practice according to domain. Besides quartile 3 in the household domain and quartile 4 in the transportation domain, which showed greater chances of having depressive symptoms, quartiles 2 and 3 from the leisure-time domain and quartile 3 from the transportation domain showed a lower chance. In a recent systematic review, Singh et al. ^25^ observed a beneficial effect of physical activity for individuals diagnosed with depression. The authors argued that those individuals had a greater potential for improvement than the non-clinical populations ^25^. Hence, our findings are not in agreement with the referred review, since we observed higher associations among subjects who are not diagnosed with depression. Non-controlled factors, such as the time of diagnosis, may partially explain those results. In any case, we must reinforce that the values observed for OR in the leisure-time domain were not very different when comparing those with the depression diagnosis (OR = 0.80; 95%CI: 0.60-1.05) with those without (OR = 0.74; 95%CI: 0.62-0.87), given that the 95%CI of the group with diagnosis is broader in terms of the lower number in the sample. Furthermore, in the analysis by quartiles, quartile 2 of the leisure-time domain showed an OR even a little lower for the group with a diagnosis (OR = 0.64) than for the group without a diagnosis (OR = 0.70), considering that the relationship was significant in both cases. Further studies are necessary to better understand the specificities of the relationships between depressive symptoms and having a diagnosis or not.

When we talk about physical activity, we must understand that different elements, from personal to social and economic ones, may either help or hamper people in becoming physically active ^7^ ^,^ ^9^ ^,^ ^29^. In Brazil, over the years, access to physical activity is differentiated according to the characteristics of the individuals, such as income, educational level, sex, among others ^3^ ^,^ ^33^ ^,^ ^34^. Limiting the definition of such practice to the recommendations of duration and intensity represents a limitation in the relationship between physical activity and the different health indicators.

In addition to the paradox of physical activity, the findings of this research indicate the need to problematize the approaches used to promote physical activity, which define that physical activities are beneficial for health, a priori and independent of other elements. That approach is often exclusive and defined by parameters such as duration, intensity, energetic output, etc. In this sense, the data from this study reinforce the need to expand our view and consider other elements of physical activity, including contextual and symbolic elements.

Similar to Lopes et al. ^19^, who found non-linear associations between the physical activity domains and depressive symptoms among Brazilians, and observed specificities of that association related to age group, this study also observed important specificities regarding sex (association with transportation physical activity only among men, and with work physical activity only among women), educational level (association with transportation physical activity only among those with lower educational level, and with work physical activity more evidently among those with a higher educational level), and among those who reported or not having a diagnosis of depression, given that the leisure-time, transportation, and work domains were associated only for those who did not report a diagnosis of depression.

Among the limitations of this study, we can mention the estimation of physical activity practice in the four domains using a questionnaire, since self-reported information may have bias of under- or overestimation, even though the method is highly accepted and used with population samples. The PHQ-9 is an instrument created to screen depressive symptoms, and not diagnose it. It was previously validated for the Brazilian population ^22^ and has been widely used in epidemiological studies. The variable “self-reported depression” should be answered based on the information “have you been diagnosed with depression?”, not necessarily referring to a recent time. Nevertheless, the “treatment of depression” variables used as confounding variables refer to the ongoing treatment at the time of data collection.

Although a previous study investigated the association between the physical activity domains and depression among the Brazilian population ^19^, our study is original in the sense that it investigates specificities related to sex, educational level, and to the presence or not of self-reported depression diagnosis. The stratified results add a contribution to the understanding of the relationship between depression and physical activity in different subgroups. Another strength of this study is the fact that the sample is representative of the Brazilian population. We recommend conducting future studies that can aid in the better understanding of some of the findings in this study, such as the relationship between the transportation domain and depression, especially considering different educational levels. Moreover, cross-sectional analyses may be conducted, considering the influence of the confounding variables and the differences observed in some subgroups.

In conclusion, considering the relationship between the physical activity practice and depressive symptoms, the idea that “every move counts” needs to be relativized. On the one hand, we observed a lesser chance of depressive symptoms among those who are active in the leisure-time domain, on the other hand, those who were active in the other domains (transportation, household, and work) showed a greater chance of presenting depressive symptoms. Therefore, physical activity should not be considered only from a biological perspective, which evaluates, for instance, only the matter of energy output, especially when we deal only with outcomes, such as that reported in this study, which relates to mental health issues. In this sense, other elements, such as pleasure, satisfaction, and cultural significance must be examined as well.
